# Feasibility of volumetric MRI-guided high intensity focused ultrasound (MR-HIFU) for painful bone metastases

**DOI:** 10.1186/2050-5736-2-16

**Published:** 2014-10-10

**Authors:** Merel Huisman, Mie K Lam, Lambertus W Bartels, Robbert J Nijenhuis, Chrit T Moonen, Floor M Knuttel, Helena M Verkooijen, Marco van Vulpen, Maurice A van den Bosch

**Affiliations:** 1 Department of Radiology, University Medical Center Utrecht, Heidelberglaan 100, 3508 GA Utrecht, The Netherlands; 2 Image Sciences Institute, University Medical Center Utrecht, 3508 GA Utrecht, The Netherlands; 3 Department of Radiation Oncology, University Medical Center Utrecht, 3508 GA Utrecht, The Netherlands

**Keywords:** Focused ultrasound, Magnetic resonance imaging, Bone metastases, Palliative treatment, Oncology, Feasibility

## Abstract

**Background:**

Magnetic resonance-guided high intensity focused ultrasound (MR-HIFU) has recently emerged as an effective treatment option for painful bone metastases. We describe here the first experience with volumetric MR-HIFU for palliative treatment of painful bone metastases and evaluate the technique on three levels: technical feasibility, safety, and initial effectiveness.

**Methods:**

In this observational cohort study, 11 consecutive patients (7 male and 4 female; median age, 60 years; age range, 53–86 years) underwent 13 treatments for 12 bone metastases. All patients exhibited persistent metastatic bone pain refractory to the standard of care. Patients were asked to rate their worst pain on an 11-point pain scale before treatment, 3 days after treatment, and 1 month after treatment. Complications were monitored. All data were prospectively recorded in the context of routine clinical care. Response was defined as a ≥2-point decrease in pain at the treated site without increase in analgesic intake. Baseline pain scores were compared to pain scores at 3 days and 1 month using the Wilcoxon signed-rank test. For reporting, the STROBE guidelines were followed.

**Results:**

No treatment-related major adverse events were observed. At 3 days after volumetric MR-HIFU ablation, pain scores decreased significantly (*p* = 0.045) and response was observed in a 6/11 (55%) patients. At 1-month follow-up, which was available for nine patients, pain scores decreased significantly compared to baseline (*p* = 0.028) and 6/9 patients obtained pain response (overall response rate 67% (95% confidence interval (CI) 35%–88%)).

**Conclusions:**

This is the first study reporting on the volumetric MR-HIFU ablation for painful bone metastases. No major treatment-related adverse events were observed during follow-up. The results of our study showed that volumetric MR-HIFU ablation for painful bone metastases is technically feasible and can induce pain relief in patients with metastatic bone pain refractory to the standard of care. Future research should be aimed at standardization of the treatment procedures and treatment of larger numbers of patients to assess treatment effectiveness and comparison to the standard of care.

## Background

Pain due to bone metastases is a common clinical problem in cancer patients [1-3]. The primary palliative treatment for patients with painful bone metastases is external beam radiation therapy [4], which leads to effective pain control in around 60%–74% of patients [5]. However, pain relief is often temporary as about 50% of responders will be confronted with recurrent pain within a year [6]. In case of non-responding or relapsing pain, repeat radiation therapy is effective in 28%–68% of patients [7-9]. As an increasing proportion of patients with painful bone metastases are insufficiently palliated by radiation therapy alone, additional palliative treatments are needed in order to maintain patients’ quality of life [7,9].

An emerging non-invasive treatment modality is magnetic resonance-guided high intensity focused ultrasound (MR-HIFU) [10-14]. In MR-HIFU, focused ultrasound beams are combined with real-time magnetic resonance (MR) imaging monitoring to perform controlled thermal ablation [15,16]. Much experience has been gained with the treatment of uterine fibroids, a benign condition of the uterus for which MR-HIFU has become an increasingly accepted treatment option [10,17-19].

In oncology, one of its applications is the palliative treatment of painful bone metastases through periosteal nerve ending ablation [20-23]. This is expected to have faster onset of pain relief compared to the standard of care. A recently published sham-randomized phase III trial has established the efficacy of MR-HIFU as a secondary palliative treatment option for painful bone metastases [24].

Traditionally, MR-HIFU treatments consist of multiple single focal point sonications, referred to as point-by-point ablation [25-27]. In 2009, an MR-HIFU system with volumetric ablation capabilities was CE-marked (Conformité Européenne) for the treatment of uterine fibroids [17]. In volumetric ablation, the focal spot is electronically steered along multiple concentric circles of increasing diameter [25,19]. Volumetric ablation is expected to be more energy efficient compared to point-by-point ablation in uterine fibroids [17]. In 2011, this system was CE-marked for the treatment of painful bone metastases [21]. The purpose of this observational cohort study was to evaluate volumetric MR-HIFU ablation for painful bone metastases on three levels: technical feasibility, safety, and initial effectiveness. To our knowledge, this study represents the first evaluation of volumetric MR-HIFU for painful bone metastases in humans.

## Materials and methods

### Ethics statement

For this study, approval from the institutional review board of the University Medical Center Utrecht (Utrecht, the Netherlands) was obtained. All participants were counselled on the nature of the procedure, and all provided written informed consent for the treatment and use of their (anonymized) data for this study.

### Patient selection

Patients were referred to our academic tertiary care center (University Medical Center Utrecht, The Netherlands) for clinical MR-HIFU treatment of painful bone metastases between April 2011 and July 2013. All patients had exhausted maximal radiotherapy and analgesic treatment options for their painful bone metastasis. For inclusion, the pain arising from the index lesion at baseline had to be self-rated by the patient as ≥4 on an 11-point numerical rating scale (NRS) from 0 (no pain) to 10 (worst imaginable pain) [28]. Exclusion criteria were the presence of >3 painful bone metastases; metastases located in joints, spine, sternum, or skull; contraindications to MR imaging or procedural sedation and analgesia (PSA); presence of a potentially unstable fracture at the site of the index lesion; and lesion inaccessibility (≤1 cm distance between the index lesion and major nerves, joints, blood vessels, or organs). Patients were also excluded if the origin of the pain could not be confirmed or if the patient was considered too ill to undergo treatment. Before treatment, all patients underwent clinical examination, conventional x-ray (OmniDiagnost Eleva, Philips Healthcare, Best, The Netherlands) and diagnostic MR imaging (1.5 T Achieva, Philips Healthcare, Best, The Netherlands). The x-ray settings were approximately 80 KeV and 25 mAs. The MRI protocol included T1-weighted (T1W) turbo spin echo (TSE) scans in two orientations, a T2-weighted (T2W) scan with spectral presaturation inversion recovery (SPIR) fat suppression, and a fat-suppressed T1W (SPIR) scan in two orientations after intravenous administration of a gadolinium-based contrast agent, gadobutrol (Gadovist, Bayer Pharma AG, Berlin, Germany, 0.1 mmol/kg). X-rays and MR images were evaluated by a radiologist to determine the location, integrity of the cortical bone and lesion type (lytic, blastic, or mixed), dimensions, mechanical stability, contrast enhancement, and treatment accessibility of the target lesion. Final treatment eligibility was determined in a multidisciplinary setting.

### MR-HIFU system

Treatments were performed using a clinical MR-HIFU system (Sonalleve, Philips Healthcare, Vantaa, Finland), integrated into a 1.5-T MR scanner (Achieva, Philips Healthcare, Best, The Netherlands) [19]. The high intensity focused ultrasound (HIFU) tabletop harbors a 256-element phased array HIFU transducer (focal length of 140 mm, operating at 1.2 MHz). Specific to this system is the concept of volumetric ablation; in a homogeneous medium, in a single exposure of ultrasound (sonication), the ultrasound focus generates ellipsoidal ablated volumes (treatment cells) with a cross-sectional operator-chosen diameter of 2, 4, 8, or 12 mm. For the bone application, the corresponding fixed sonication durations for these treatment cells were 16, 16, 20, and 36 s, respectively. Volumetric ablation is realized by electronic steering of the focal spot along concentric circles of increasing diameter, except for the 2 mm treatment cell which is the result of a single sonication of a static beam. Maximal acoustic power levels ranged from 190 W for the smallest treatment cell to 80 W for the largest treatment cell. The maximal sonication energy was 3.0 kJ.

### Treatment preparation

Before treatment, the skin overlying the site of interest was shaved and auricular temperature was measured. Premedication consisted of 10 mg dexamethasone/8 mg ondansetron intravenous. Patients were positioned on the HIFU tabletop with the point of maximum pain above the transducer. Acoustic coupling between the transducer and the patient was facilitated through one or more gel pads (1.5 cm thickness, Aquaflex, Parker Laboratories, Fairfield, USA) moistened with degassed water.

The first four patients received moderate PSA (‘conscious sedation’) using 50–100 μg fentanyl/2–5 mg midazolam intravenous; the remaining patients received deep PSA (‘deep sedation’) administered by a PSA specialist using propofol intravenous (induction 0.5–1 mg/kg, maintenance 5 mg/kg/hour) combined with analgesics at the discretion of the PSA specialist. The patients’ vital functions were constantly monitored and patients were visually observed using a multi-camera observation system. Positioning and treatment planning were verified using multi-planar reconstructions of three-dimensional T1W spoiled-gradient echo scans.

### Ablation method

**Figure 1:**
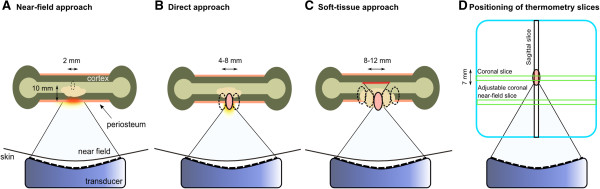
**Ablation approach and thermometry slice positioning. (A)** Near-field approach: in lesions with (partially) intact cortical bone, treatment cells were positioned 10 mm distal to the cortical bone to generate temperature increase along the cortical surface that intersects the beam path. **(B)** Direct approach: the treatment cells were positioned on the cortical surface. **(C)** Soft-tissue approach: in lytic lesions exhibiting complete cortical destruction, treatment cells were positioned within the tumor mass and its close proximity to achieve ablation of possible remaining periosteal nerves and the tumor itself. **(D)** Position of thermometry slices: the position of the slices relative to the treatment cell (red) is fixed except for the coronal near-field slice.

The optimal ablation approach (i.e., energy deposition method) has not been established yet for volumetric MR-HIFU ablation of painful bone metastases. In patients with (partially) intact cortical bone, two different ablation approaches were used to ablate the periosteum, i.e., the near-field approach and the direct approach [29]. In the *near-field approach*, treatment cells were positioned 10 mm distal to the cortical bone to generate temperature increase along the cortical surface intersecting the beam path (Figure 1A). In the *direct-treatment approach*, the treatment cell was positioned on the cortical surface (Figure 1B). The main factors determining choice of ablation approach were safety concerns. In both the near-field and the direct approach, the beam path was positioned as perpendicular as possible to the cortical surface while avoiding critical structures. In case of complete cortical destruction the cortical bone-soft-tissue interface could not be targeted; the treatment cells were positioned in the tumor mass itself to achieve ablation of possible remaining periosteal nerves and the tumor (Figure 1C).

**Figure 2:**
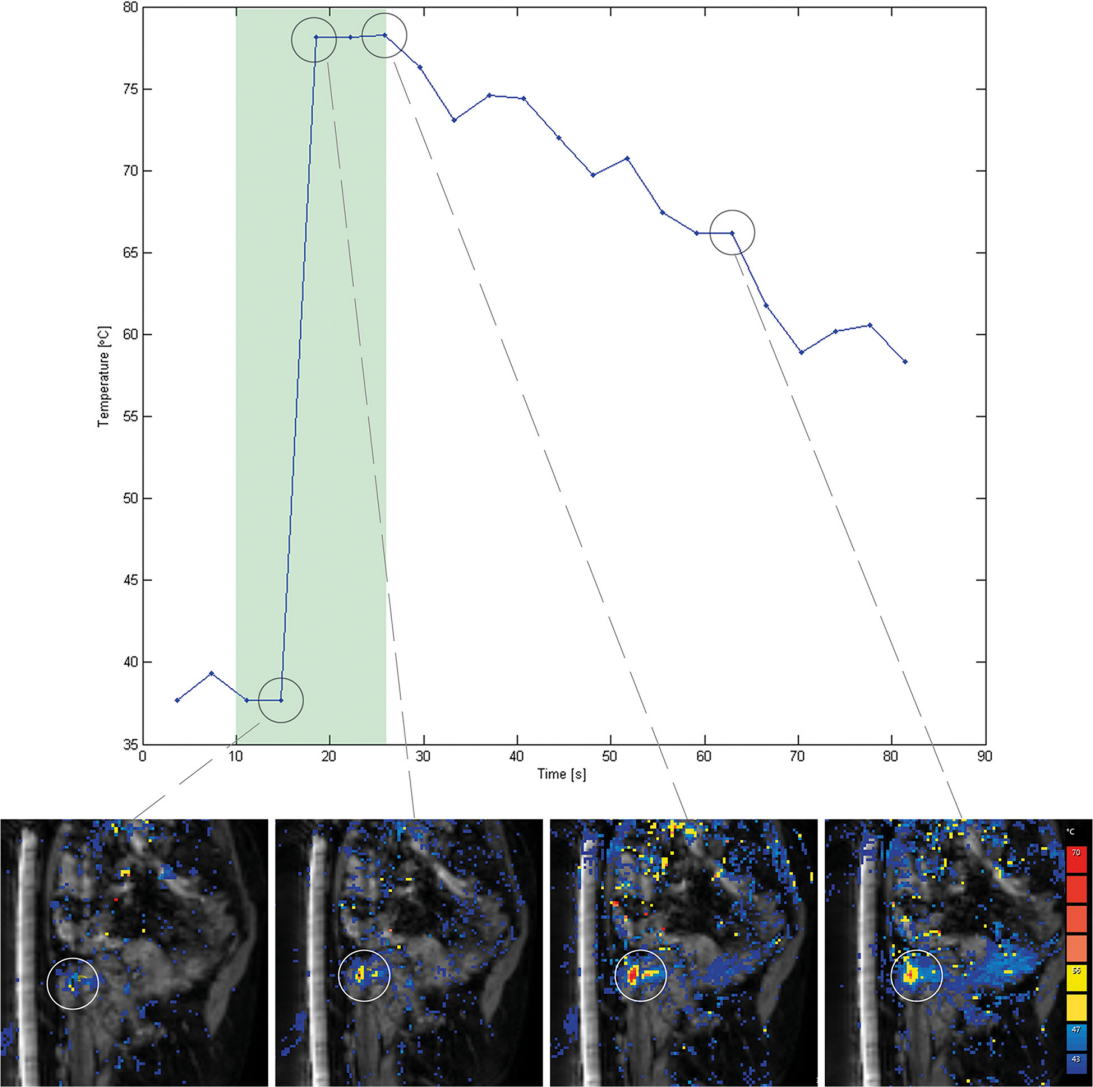
**Example of temperature increase during sonication.** The graph displays the maximal temperature reached in the target area during a therapeutic sonication in a bone metastasis in the pubic bone with a partially intact cortex (near-field approach). The green area indicates the duration of sonication. The values displayed are based on the voxel with the highest temperature. The sagittal magnitude images with temperature color map overlays show the temperature increase at the bone-soft-tissue interface (encircled in white); orange and red colors indicate a temperature over 56°C.

Treatment cell diameters were determined according to target sizes and treating physician (MB, RN, and MH) preferences. During each sonication, temperature differences with respect to the baseline body temperature of the patient were measured using the proton resonance frequency shift (PRFS) MR thermometry method [30]. Temperature color maps were overlaid onto the magnitude images acquired using the thermometry scan on the HIFU treatment console. Since cortical bone generates virtually no MR signal due to its extremely short transverse relaxation time, thermal mapping is only possible by measuring temperature changes occurring in the soft tissue adjacent to the cortical bone. The thermometry sequence was a fast field echo (FFE) with echo planar imaging (EPI) with four slices: one coronal slice perpendicular to the beam through the center of the treatment cell, one sagittal slice along the central axis of the beam path through the center of the treatment cell, one transverse slice along the central axis of the beam path through the center of the treatment cell, and one adjustable coronal near-field slice (Figure 1D). The generally used parameters were as follows: effective echo time 19 ms, repetition time 36 ms, flip angle 20°, EPI factor 9, voxel size 2.5 × 2.5 × 7 mm^3^, FOV 400 × 310 mm^2^, number of signal averages (NSA) 2, and temporal resolution 3.7 s. Water-selective binomial RF excitation pulses (1-2-1) were used. Prior to treatment planning, a temperature scan was acquired to identify any artifacts likely to occur during treatment, for example, bowel motion artifacts. At least one test sonication per patient (20–40 W; 0.3–0.6 kJ) was performed. The therapeutic acoustic power level was determined by the treating physician. An ablation was considered successful when the temperature mapping indicated a temperature above the threshold of 55°C at the level of the target [31,32]. Entire lesion surface coverage was aimed for by systematically sonicating one or multiple rows of treatment cells in an interleaved fashion. All sonications were analyzed to identify motion in the target area and to evaluate the changes in temperature over time. An example is given in Figure 2. The maximal temperature reached in the target area was recorded for each sonication; thermal maps disturbed by patient motion were not used for analysis.

### Follow-up and response assessment

All data were prospectively recorded in the context of routine clinical care. Prior to treatment, 3 days after treatment, and 1 month after treatment, patients were asked to rate their worst pain in the past 3 days on an 11-point scale (NRS). At the same time points, their analgesic use over the past 24 h was recorded and possible complications were monitored. Further follow-up was done as clinically indicated. Partial response was defined as either a ≥2-point decrease in NRS at the treated site without increase in daily oral morphine equivalent (OMED) or as OMED reduction of 25% or more from baseline without an increase in pain. Complete response was defined as a pain score of 0 at the treated site with no concomitant increase in OMED. Pain progression was defined as a ≥2-point increase at the treated site with stable OMED or an increase of 25% or more in OMED compared with baseline with the pain score stable or 1 point above baseline. These definitions are in accordance with previously established standards as published by the International Bone Metastases Consensus Working Party [33,34].

### Statistical analysis

Categorical data are summarized as frequencies and proportions, continuous variables are summarized as means or medians with (interquartile) range, depending on their distributions. Overall response rate with corresponding confidence interval (CI, adjusted Wald method) was calculated as the proportion of responders of evaluable patients. Wilcoxon signed-rank test was used to compare baseline pain scores and pain scores at 3 days and 1 month after treatment. In case of multiple treatments per patient, only the first treatment was used for pain response calculation. Analyses were performed using IBM SPSS Statistics, version 20.0 (Armonk, NY, USA).

## Results

Twenty-four (*n* = 24) patients were referred for MR-HIFU treatment and screened for eligibility. Eleven patients (46%) were considered eligible and underwent MR-HIFU treatment. The remaining 13 patients were ineligible for the following reasons: index lesion was not the origin of pain (*n* = 5), mechanical instability (*n* = 2), inaccessible lesion (*n* = 3), and poor clinical status (*n* = 3). Eleven consecutive patients (7 male and 4 female; median age, 60 years; age range, 53–86 years) underwent 13 treatments for 12 bone metastases. Baseline characteristics of these patients are presented in Table 1. Most lesions were lytic (7/12, 58.3%), four lesions (33.3%) were mixed and one lesion (8.3%) was blastic. The majority of patients had lesions with at least part of the cortical bone intact (7/12, 58%), and the cortical bone was completely destructed by the tumor in five patients (42%).

**Table 1:** Patient and lesion characteristics

**Patient**	**Sex**	**Age (years)**	**ECOG ps**	**Primary tumor**	**Location**	**Lesion type**	**Cortex**^**c**^	**Lesion dimensions (mm)**^**d**^
1 lesion a^a^	M	58	1	Kidney	Femur	Lytic	Complete destruction	69 × 63 × 131
1 lesion b^a^	M	58	1	Kidney	Femur	Lytic	Complete destruction	84 × 52 × 100
2	F	55	1	Kidney	Sacrum	Lytic	Complete destruction	64 × 27 × 64
3	F	56	1	Colorectal	Pubic bone	Mixed	Partially intact	59 × 22 × 23
4	M	60	1	Colorectal	Pubic bone	Lytic	Complete destruction	62 × 36 × 45
5^b^	F	64	0	Breast	Sacrum	Mixed	Intact	107 × 56 × 56
6	F	53	2	Breast	Humerus	Blastic	Intact	36 × 28 × 23
7	M	86	1	Sarcoma	Rib	Mixed	Partially intact	56 × 39 × 46
8	M	55	1	Prostate	Pubic bone	Lytic	Partially intact	64 × 44 × 45
9	M	71	1	Lung	Pubic bone	Lytic	Complete destruction	69 × 48 × 75
10	M	65	2	Colorectal	Rib	Mixed	Partially intact	122 × 29 × 29
11	M	64	2	Mesothelioma	Rib	Lytic	Partially intact	74 × 9 × 6

*Abbreviations*: *ECOG ps* Eastern Cooperative Oncology Group performance score.

^a^Patient 1 was treated on two locations: 1a midshaft left femur and 1b distal left femur.

^b^Patient 5 was retreated on the same location 4 months after the first treatment because of recurrent pain.

^c^Appearance on x-ray or CT-imaging (if available) before treatment: intact = no or minimal architectural distortion of the cortical bone by the tumor; partially intact = both destructed and intact cortical bone in the targeted area; complete destruction = cortical bone completely displaced by tumor.

^d^Long axis (transversal) × short axis (transversal) × craniocaudal dimension (coronal) as measured on pretreatment CE-MRI.

**Figure 3:**
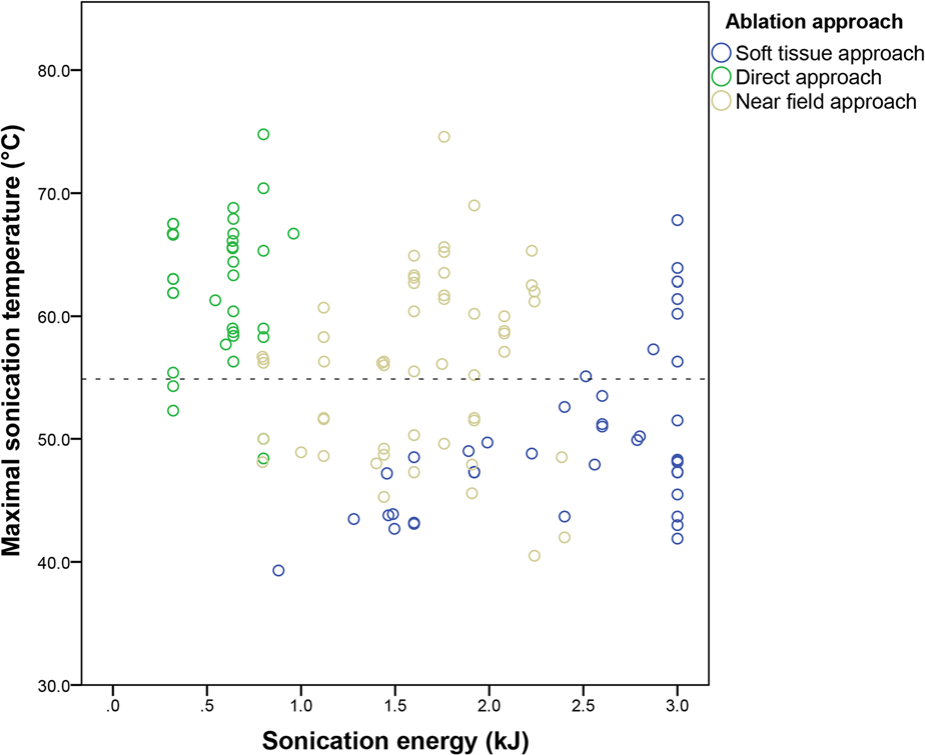
**Sonication energy, temperature, and ablation approach.** Scatterplot of sonication energy (kJ) on the *x*-axis and temperature (°C) on the *y*-axis, stratified per ablation approach (legend). Each dot represents a single sonication. The horizontal dotted line indicates 55°C. The measured temperatures for the direct approach (green) are mostly over 55°C at low sonication energies. For the near-field approach (grey), measured temperatures were both above and below 55°C (even with energies around 2.0 kJ). In lytic lesions with complete cortical destruction in which soft-tissue ablation was aimed for (blue), measured temperatures were also below and above 55°C (even at energies of 3.0 kJ).

Of 13 treatments, four treatments were performed using the *near-field approach* and three using the *direct approach*. In the five lesions exhibiting complete cortical destruction, soft-tissue ablation of the tumor was aimed for. Detailed treatment parameters, including treatment cell diameter, are listed in Table 2. The median treatment time (from first sonication to the last) was 45 min (range 20–73). Pain or discomfort was reported by all three patients treated under moderate PSA and only by one of eight patients treated under deep PSA.In the first patient, the second treatment was terminated early due to the presence of severe susceptibility artifacts caused by close proximity of titanium fixation screws, more pronounced than during the first treatment. In one patient with a rib lesion and a discontinuous cortex, the treatment cells were positioned in the tumor mass just in front of the rib to avoid potential thermal damage of the lung in the far field. Sonication energies needed to reach the targeted temperature were lowest for the direct approach (median 0.6 kJ, range 0.3–1.0), compared to the near-field approach (median, 1.7 kJ, range 0.2–2.4) and soft-tissue ablation (median 2.4 kJ, range 0.9–3.0). Measured temperatures were highest for the direct approach (median 63.0°C, range 48.4°C–74.8°C) compared to the near-field approach (median 56.3°C, range 40.5°C–74.6°C). In patients in whom soft-tissue ablation was aimed for, temperatures above 55°C were difficult to achieve with the current software (median 48.3°C, range 39.3°C–67.8°C) (Figure 3).

**Table 2:** Treatment parameters (chronological order)

**Treatment**	**Duration (min)**	***N *** **treatment cells (*n *** **sonications)**	**Treatment cell diameter (mm)**	**Acoustic power (W) median, range**	**Sonication energy (kJ) median, range**	**Total delivered energy (kJ)**	**Temperature (°C) median, range**	**Ablation approach**^**b**^
1a^a^	40	2 (10)	4	100 (40–140)	2.0 (0.8–2.8)	17.5	-	Soft tissue
1b^a^	20	3 (6)	2	50 (40–60)	1.0 (0.8–1.2)	6.0	-	Soft tissue
2	41	5 (6)	2, 4	80 (50–100)	1.5 (0.9–1.9)	8.8	43.9 (39.3–49.0)	Soft tissue
3	37	6 (10)	4	110 (90–120)	1.8 (1.1–1.9)	16.6	63.1 (56.3–74.6)	Near field
4	45	7 (15)	4, 8	120 (60–160)	2.2 (1.0–3.0)	31.3	47.3 (43.1–50.2)	Soft tissue
5a	37	10 (11)	4	50 (50–70)	0.8 (0.8–1.1)	10.2	56.2 (48.1–60.7)	Near field
6	73	18 (28)	4	100 (50–130)	1.6 (0.6–2.1)	42.4	55.2 (45.3–60.4)	Near field
7	49	16 (19)	4	130 (70–150)	2.1 (1.1–3.0)	39.1	64.8 (51.3–75.5)	In front of cortex^c^
5b	69	20 (22)	4,8	40 (30–50)	0.6 (0.4–1.0)	14.9	60.9 (48.4–70.9)	Direct
8	68	22 (25)	4	120 (90–150)	1.9 (1.4–2.4)	48.4	56.1 (40.5–65.6)	Near field
9	71	21 (22)	8, 12	150 (70–150)	3.0 (2.0–3.0)	62.6	51.4 (41.9–67.8)	Soft tissue
10	39	9 (15)	4	20 (20–20)	0.3 (0.3–0.3)	4.2	63.0 (52.3–67.5)	Direct
11	45	12 (12)	4	40 (40–60)	0.6 (0.6–1.0)	7.4	64.4 (58.4–74.8)	Direct

*Abbreviations*: °C degrees Celsius, *mm* millimeter, *min* minutes, *kJ* kilojoule, *W* watt, *CR* complete response, *PR* partial response, *NR* no response.

^a^In treatment 1a and 1b, no representative temperature data were available due to susceptibility artifacts caused by internal fixation material. Treatment 1b was terminated early due to severe susceptibility artifacts (targeted volume too close to fixation material).

^b^Direct approach: the treatment cell was positioned on the cortical surface. Near-field approach: the treatment cells were positioned 10 mm distal to the cortical bone. Soft-tissue approach: in lesions with complete cortical destruction, the treatment cells were positioned in the tumor mass itself.

^c^Because of lungs in the far field, for safety reasons, treatment cells were positioned in the tumor mass in front of the cortical bone.

### Pain response and complications

**Figure 4:**
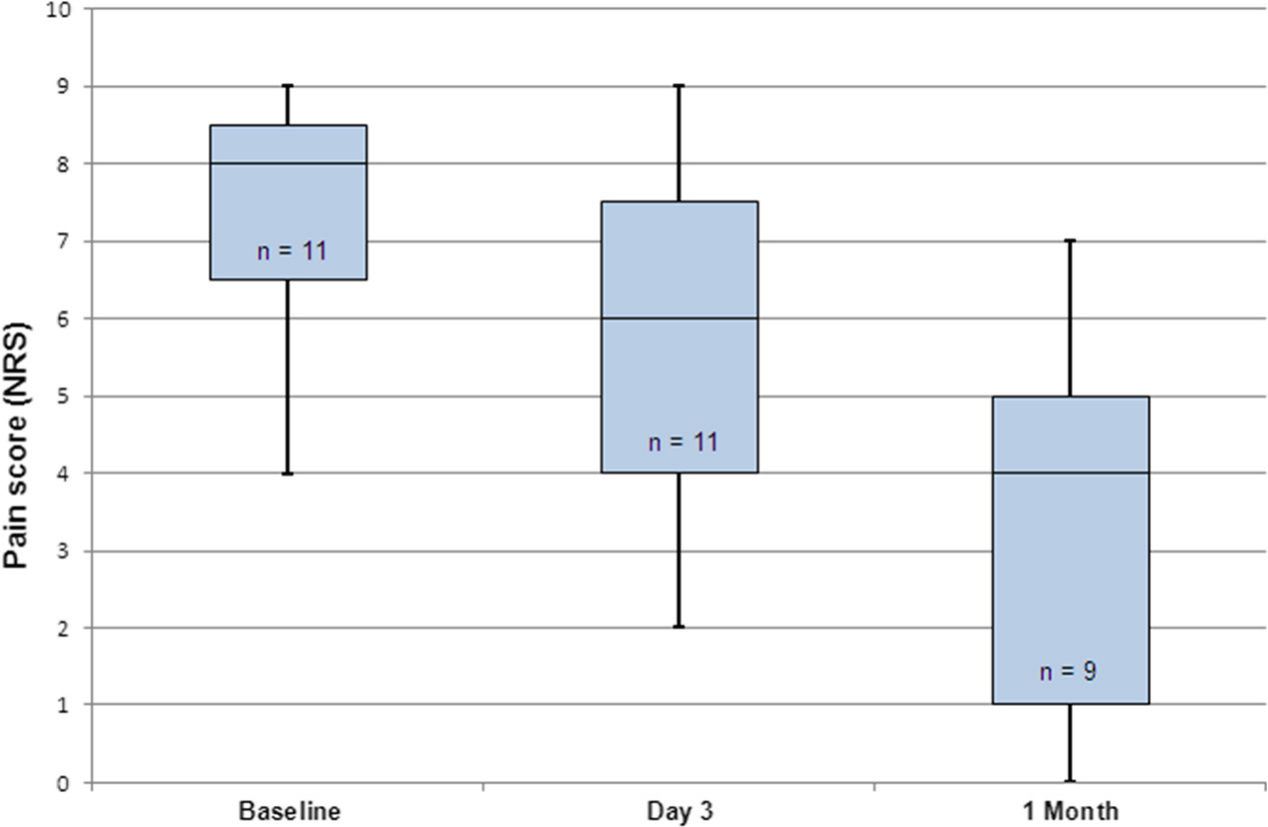
**Pain response.** Boxplot of pain scores before treatment, 3 days after treatment, and 1 month after treatment. In the graph, the median is shown (middle black line), minimum and maximum values (bars), and lower and upper quartiles (boxes).

No treatment-related major complications during or after treatment were seen. Minor complications included pain after treatment (*n* = 1) and a first degree skin burn (*n* = 1). All patients could be discharged on the day of treatment. Three days after treatment, the pain score decreased significantly (*p* = 0.045) from baseline median of 8 (1st-3rd quartile 6–9) to 6 (1st-3rd quartile 4–8, Figure 4). Partial response was observed in six patients (6/11, 55%) and pain progression in one patient (9%). One-month follow-up was available for nine of eleven patients (82%). One patient died 3 weeks after treatment due to his primary disease; the other patient withdrew from follow-up 3 weeks after treatment due to progressive systemic disease. At 1 month follow-up, the pain score decreased significantly compared to baseline pain scores (median 4, 1st-3rd quartile 1–5, *p* = 0.028, Figure 4). At 1 month, five patients (5/9, 56%) showed partial response and one patient (1/9, 11%) showed complete response. There were no cases of pain progression at 1 month. The overall response rate was 67% (95% CI 35%–88%). In Table 3, the pain scores (NRS) and changes in analgesic intake (OMED) are presented per patient. The mean duration of clinical follow-up was 8.7 weeks (range 2.9–24.0).

**Table 3:** Changes in pain score, analgesic intake, and response

**Treatment**	**Pain scores (NRS)**			**Analgesic intake**^**c**^	**Response**^**d**^
	**Baseline**	**Day 3**	**1 Month**		
1a	7	4	4	Stable	PR
1b^a^	7	7	7	Stable	NR
2	8	8	7	Stable	NR
3	8	9	2	Stable	PR
4	6	6	7	Stable	NR
5a	8	8	4	Reduction	PR
6	9	4	0	Increase	PR
7	4	2	5	Stable	NR
5b^a^	10	8	5	Reduction	PR
8	9	7	0	Stable	CR
9	6	6	1	Reduction	PR
10^b^	9	4	-	-	**-**
11^b^	7	7	-	-	**-**

*Abbreviations*: *NRS* numeric rating scale.

^a^Second treatment in the same patient was not included in the response analysis.

^b^No follow-up data available at 30 days after treatment, only included in response analysis at 3 days.

^c^At 1 month after treatment compared to baseline; reduction is defined as ≥25% decrease in daily oral morphine equivalent (OMED), increase is defined as ≥25% increase in OMED, and stable is defined as neither increase nor decrease [33,34].

^d^Response at 1 month as defined by the updated International Bone Metastases Consensus Working Party [33,34]. Partial response (PR): pain reduction of ≥2 or more at the treated site on a 0 to 10 scale without analgesic increase or analgesic reduction without increase in pain. Complete response (CR): pain score of 0 at the treated site with no concomitant increase in analgesics. No response (NR): no partial or complete response.

## Discussion

To the best of our knowledge, this is the first study reporting on volumetric MR-HIFU ablation for painful bone metastases.

No treatment-related major adverse events were observed during the study period. The results of our study showed that volumetric MR-HIFU ablation for painful bone metastases is technically feasible and can induce pain relief in patients with metastatic bone pain refractory to the standard of care. Three days after treatment, the pain score decreased significantly compared to baseline and partial response was observed in six of eleven patients. At 1 month follow-up, pain scores were significantly lower than at baseline and pain response was obtained in six of nine patients. The results of our study are in line with the work of others and support the conclusion that MR-HIFU could be a safe and effective palliative treatment option for patients with intractable metastatic bone pain [20,22,35,24]. The early onset of response might be an advantage compared to radiation therapy in which response is typically seen after 3 to 4 weeks [6].

During the course of this study, several technique modifications were introduced. In the early treatments, relatively few treatment cells and lower powers were used compared to later treatments. Apart from the natural learning curve, pain and discomfort during treatment under moderate PSA limited the use of higher acoustic power levels. Hence, some early patients may have been undertreated. The use of deep PSA has considerably improved patient comfort and treatment tolerability and this experience might be useful for other physicians who want to adopt the treatment.

As the optimal treatment strategy for volumetric MR-HIFU treatment of painful bone metastases has not been established yet, several ablation approaches were used. In patients with (partially) intact cortical bone at the targeted volume, treatment cells were initially positioned behind the cortical bone (near-field approach), and in later treatments, treatment cells were positioned on the bone-soft-tissue interface (direct approach). Although both ablation approaches are able to induce thermal ablation of the bone-soft-tissue interface, the direct approach requires lower sonication energies compared to the near-field approach minimizing the risk of thermal damage beyond the targeted volume [29]. This was also observed in our study. Also, thermal mapping in the coronal plane generally was of higher quality in the direct ablation approach due to the fixed alignment of the thermometry slices relative to the treatment cell.

In lesions exhibiting complete cortical destruction, a different treatment strategy may be required with regard to treatment cell positioning and choice of acoustic power level. For true soft-tissue ablation and debulking, much like in uterine fibroids for example [36], higher sonication energies (>3.0 kJ) are probably necessary.

Since lethal cell damage occurs when temperatures >55°C are maintained for longer than a second [31,32], accurate temperature measurements are crucial to monitor the treatment and to evaluate treatment outcomes. In this small patient group, temperatures above the threshold of 55°C were not reached in every patient, especially in those with lesions exhibiting complete cortical destruction. These were also the ones that did not show a treatment response. This may also be attributable to the fact that in lesions exhibiting complete cortical destruction the periosteum cannot easily be targeted and might not even be present anymore. There was one other non-responder in which periosteal ablation is not likely to have occurred since the treatment cells were placed in the tumor mass in front of the (partially intact) cortex for safety reasons.

In MR-HIFU, image guidance is extremely important for both treatment planning and real-time temperature monitoring. Although PRFS-based thermometry is currently the most common MR thermometry method, there are some challenges with regard to its application in bone treatments. First, only temperature differences occurring in aqueous soft-tissue adjacent to the cortical bone can be measured [37]. Second, the method is particularly sensitive to magnetic field disturbances and artifacts [38,39]. Lastly, partial volume effects may introduce some inaccuracy in temperature estimates [39]. Therefore, it must be noted that the measured temperatures only represent an approximation of the true temperature of the bone-soft-tissue interface even though thermal maps disturbed by patient motion were not used for analysis in this study.

This study has several limitations; the most important limitation is the fact that the studied patient population was rather small and heterogeneous and that the follow-up period was relatively short. Also, this initial experience with the volumetric MR-HIFU treatment for painful bone metastases does not allow for conclusive statements regarding the most effective treatment strategy. Nonetheless, we believe that this study provides important information for others wanting to perform this treatment, and its results may serve as a basis for further research. Furthermore, imaging outcomes were not incorporated in this study, as follow-up by imaging was not part of our routine clinical care. Imaging might be relevant when looking at treatment outcomes, as reported by others [20,23,35]. As a pretreatment computed tomography (CT) scan was not part of the study protocol, a pretreatment CT scan was only available in some patients. It was found that a CT scan has an added value to x-ray and MR imaging alone, as precise information on the integrity of the cortical bone is important for treatment planning.

Despite these limitations, this study represents the first experience with volumetric MR-HIFU ablation for palliation of painful bone metastases. Consecutive report and in-depth evaluation of a new technique is essential for its development [40]. If MR-HIFU is to be translated to clinical practice as a serious competitor to the standard of care, standardization of the treatment methodology is key [40]. This study provides a basis for further development and standardization of the technique. In addition to technical development and preclinical research, future research should include a well-designed large cohort study with longer follow-up, in which patients with persistent metastatic bone pain are treated. The results of this study could strengthen the rationale for comparative studies for example radiotherapy versus MR-HIFU or radiotherapy in combination with MR-HIFU versus radiotherapy alone. In conclusion, no major treatment-related adverse events were observed during follow-up, and this study showed that volumetric MR-HIFU ablation of painful bone metastases is technically feasible and can induce pain relief in patients with metastatic bone pain refractory to the standard of care. Future research should be aimed at optimization and standardization of the technique and treatment of larger patient populations with longer follow-up to establish treatment effectiveness and comparison with the standard of care.

## Competing interests

The authors declare that they have no competing interests.

## Authors' contributions

MH conducted the study, participated in its design, performed the statistical analysis and drafted the manuscript. ML participated in conduct of the study and data collection. LB participated in the design and conduction of the study. RN supervised clinical treatments and participated in analysis. CM participated in the design and conduction of the study. FK participated in data collection. HV participated in the design of the study and analysis. MV participated in the design of the study. MB initated and supervised the study as well as clinical treatments. All authors read and approved the final manuscript..
